# Laser-activated irrigation versus ultrasonic or syringe irrigation for smear layer and debris removal: An *in vitro* SEM study

**DOI:** 10.6026/973206300222040

**Published:** 2026-04-30

**Authors:** Nikita Aji Mannur, Saloni Bharti, Monica Sinha, Arisha Jain, Rishikesh Meshram

**Affiliations:** 1 Department of Conservative Dentistry and Endodontics, SJM Dental College & Hospital, Chitradurga, Karnataka, India; 2 Department of Dentistry, ESIC Medical College and Hospital, Kateshar, Bihta, Bihar, India; 3 Department of Prosthodontics, Dental Care Clinic Palika Health Complex, Chanakyapuri, NDMC, Delhi - 110021, India; 4 Department of Conservative Dentistry and Endodontics, Dasmesh Institute of Research and Dental Sciences, Faridkot, Punjab, India; 5 Department of Conservative Dentistry and Endodontics, Swargiya Dadasaheb Kalmegh Smruti Dental College and Hospital, Wanadongri, Hingna, Nagpur - 441110, Maharashtra, India

**Keywords:** Laser-activated irrigation (LAI), smear layer, debris removal, root canal, Er:YAG laser, endodontics, irrigation techniques

## Abstract

Effective removal of smear layer and debris is essential for predictable endodontic disinfection, yet conventional irrigation remains insufficient, particularly in the apical third. Therefore, it is of interest to compare laser-activated irrigation (LAI), passive ultrasonic irrigation (PUI) and conventional syringe irrigation (CSI) using SEM evaluation of ninety single-rooted teeth. LAI demonstrated significantly lower smear layer and debris scores across all canal thirds compared to PUI and CSI (p<0.001). Complete debris removal throughout the canal was achieved in 93.3% of LAI samples versus 73.3% for PUI and 43.3% for CSI. Thus, laser-activated irrigation produced more consistent apical cleaning, indicating superior hydrodynamic activation efficiency.

## Background:

Successful endodontic treatment depends on effective mechanical preparation combined with thorough chemical debridement of the root canal system [1]. Instrumentation inevitably produces a smear layer composed of organic tissue remnants, inorganic dentin particles and microorganisms that can obstruct dentinal tubules and compromise disinfection [2]. Persistence of smear layer and debris reduces irrigant penetration, limits sealer adaptation and may contribute to treatment failure [3]. Conventional syringe irrigation using sodium hypochlorite remains the standard approach; however, its effectiveness is limited by inadequate irrigant exchange, vapor lock formation, restricted apical penetration and inability to reach anatomical complexities [4]. Passive ultrasonic irrigation improves cleaning through acoustic microstreaming and cavitation, yet its efficacy remains inconsistent in the apical third [5]. The apical region presents the greatest challenge due to reduced canal diameter, complex anatomy and limited irrigant dynamics [6].

Laser-activated irrigation has emerged as an advanced activation technique that utilizes photoacoustic streaming and cavitation generated by laser-irrigant interaction [7]. The Er:YAG laser, characterized by high water absorption at 2940 nm, produces rapid vapor bubble expansion and collapse, generating shockwaves capable of enhancing irrigant agitation and penetration [8]. Therefore, it is of interest to compare the effectiveness of laser-activated irrigation, passive ultrasonic irrigation and conventional syringe irrigation in removing smear layer and debris from different root canal thirds using standardized SEM evaluation.

## Materials and Methods:

This *in vitro* experimental study was conducted on ninety freshly extracted single-rooted permanent human teeth obtained for periodontal or orthodontic reasons with informed consent for research use. Teeth were cleaned of soft tissue remnants and stored in 0.5% chloramine-T solution at 4°C for no longer than one month. Only teeth with mature apices, single straight canals (<10°C curvature), absence of prior endodontic treatment and patency with size 10 K-file to working length were included. Teeth with resorption, calcification, fractures, structural defects, complex canal anatomy, or open apices were excluded. Crowns were sectioned at the cementoenamel junction to standardize root length to 15 mm. Sample size was calculated using G*Power software (effect size 0.7, α=0.05, power=0.85), yielding a minimum of 27 specimens per group; thirty samples per group were included to compensate for potential losses. Specimens were randomly allocated into three groups (n=30) using computer-generated randomization: conventional syringe irrigation (CSI), passive ultrasonic irrigation (PUI) and laser-activated irrigation (LAI). All canals were prepared by a single experienced operator using ProTaper Universal rotary instruments up to size F3 (30/.09) at 300 rpm and 2 Ncm torque. During instrumentation, 2 mL of 5.25% sodium hypochlorite was used between files with a 30-gauge side-vented needle placed 2 mm short of working length. Final irrigation protocols were performed according to group allocation. CSI received 10 mL of 5.25% NaOCl over 3 minutes followed by 5 mL of 17% EDTA for 2 minutes and a final flush with distilled water. PUI received identical irrigants activated ultrasonically at 30 kHz using a size 15 file positioned 2 mm short of working length for three 20-second cycles per irrigant. LAI received the same irrigant sequence activated with an Er:YAG laser (50 mJ, 20 Hz, very short pulse mode) using a 400 µm fiber tip positioned 3 mm short of working length with four 15-second activation cycles. Following irrigation, specimens were split longitudinally and prepared for scanning electron microscopy. Samples were dehydrated in graded ethanol, critically point dried, sputter-coated with gold-palladium and examined under SEM at 1000x and 2000x magnification. Coronal, middle and apical thirds were evaluated. Two blinded calibrated examiners performed scoring using modified Torabinejad criteria for smear layer and standardized debris scoring. Inter-examiner reliability was assessed using Cohen's kappa coefficient. Data were analyzed using SPSS version 26.0. Normality was assessed using Shapiro-Wilk test. Kruskal-Wallis test with post-hoc Mann-Whitney U tests (Bonferroni correction) compared groups. Friedman test evaluated intragroup canal third differences. Spearman correlation assessed association between smear layer and debris scores. Statistical significance was set at p<0.05.

## Results:

All ninety specimens were successfully prepared and analyzed without procedural loss. Inter-examiner reliability was excellent for smear layer scoring (κ=0.87) and debris scoring (κ=0.84), indicating strong agreement. Smear layer scores differed significantly among the three irrigation techniques across all canal thirds (p<0.001). Laser-activated irrigation consistently demonstrated the lowest mean smear layer scores, followed by passive ultrasonic irrigation and conventional syringe irrigation. The greatest differences were observed in the apical third, where CSI showed the highest smear layer retention. Within each group, smear layer removal efficacy decreased significantly from coronal to apical thirds (p<0.001). However, LAI exhibited the smallest variation among canal thirds, indicating more uniform cleaning throughout canal length. In the coronal third, 63.3% of LAI samples achieved complete smear layer removal (Score 1) compared to 23.3% in PUI and 6.7% in CSI. In the apical third, complete smear layer removal was achieved in 26.7% of LAI samples, whereas CSI failed to achieve Score 1 in any apical specimen. Debris removal followed a similar pattern to smear layer removal. LAI demonstrated significantly lower mean debris scores across all canal thirds compared to PUI and CSI (p<0.001). Complete debris removal throughout all canal thirds was observed in 93.3% of LAI samples, compared to 73.3% in PUI and 43.3% in CSI. In the apical third, LAI achieved complete cleaning in 46.7% of specimens, whereas PUI and CSI achieved 23.3% and 6.7%, respectively. A strong positive correlation was identified between smear layer and debris scores (Spearman's ρ = 0.82, p<0.001), indicating that techniques effective for smear layer removal were similarly effective for debris elimination. Qualitative SEM observations confirmed quantitative findings, with LAI specimens demonstrating predominantly open dentinal tubules and minimal surface coverage, while CSI specimens showed thick smear layer accumulation and debris retention, particularly in the apical region. Table 1 (see PDF) shows significantly lower smear layer scores for LAI compared to PUI and CSI across coronal, middle and apical thirds (p<0.001), with the greatest improvement observed in the apical region. Table 2 (see PDF) demonstrates that 63.3% of LAI specimens achieved complete smear layer removal in the coronal third and 26.7% in the apical third, whereas CSI failed to achieve complete apical cleaning in any specimen. Table 3 (see PDF) shows significantly lower mean debris scores for LAI across all canal thirds (p<0.001), with complete debris removal observed in 93.3% of LAI specimens overall and 46.7% in the apical third, compared to 43.3% and 6.7% for CSI, respectively.

## Discussion:

This study demonstrated that laser-activated irrigation using the Er:YAG laser produced significantly superior smear layer and debris removal compared to passive ultrasonic irrigation and conventional syringe irrigation across all root canal thirds [9]. The most clinically relevant finding was the marked improvement in apical cleaning, where LAI achieved substantially higher complete debris removal rates than the other techniques [10]. These results confirm that hydrodynamic activation strength plays a decisive role in mechanical debridement efficacy [11]. The superior performance of LAI can be explained by photoacoustic streaming and secondary cavitation generated by rapid vapor bubble expansion and collapse within the irrigant [12]. The Er:YAG wavelength allows efficient energy transfer to irrigant solutions, producing three-dimensional fluid movement capable of penetrating anatomical irregularities beyond the reach of syringe needles [13]. In contrast, conventional syringe irrigation relies primarily on positive-pressure delivery, which is limited by penetration depth and vapor lock formation, particularly in the apical third [14]. Passive ultrasonic irrigation demonstrated intermediate performance, consistent with its mechanism of acoustic microstreaming and cavitation [15]. However, ultrasonic activation remains dependent on oscillation amplitude, canal wall clearance and irrigant replenishment, which may reduce effectiveness in constricted apical regions [16]. The reduced performance of PUI compared to LAI suggests that shockwave-driven irrigant activation generates stronger and more uniform canal wall shear forces [17]. The strong positive correlation between smear layer and debris removal indicates that both outcomes are governed by mechanical fluid dynamics rather than purely chemical dissolution [18]. Techniques that generate greater cavitation intensity and hydrodynamic shear stress appear to produce more comprehensive cleaning [19]. The reduced variability among canal thirds observed in the laser group further supports its ability to deliver consistent irrigant activation throughout canal length [20].

## Conclusion:

Laser-activated irrigation using the Er:YAG laser demonstrated significantly superior smear layer and debris removal compared to passive ultrasonic and conventional syringe irrigation. The technique provided more consistent cleaning across all canal thirds, with marked improvement in the apical region. Thus, laser activation represents an effective adjunctive strategy for enhancing mechanical debridement during root canal therapy.

## Advancement to knowledge:

Advancement to knowledge in this study lies in the standardized three-way comparison under controlled SEM evaluation, quantitative confirmation of apical superiority of laser activation and demonstration of consistent cleaning across canal thirds. Unlike studies focusing primarily on antimicrobial activity, this investigation specifically quantified mechanical smear layer and debris removal using validated scoring systems and correlation analysis. The *in vitro* design represents a limitation, as clinical complexity and anatomical variability may influence irrigant dynamics. Additionally, only one laser parameter configuration was tested and optimal energy settings require further investigation. Future research should evaluate clinical outcomes, long-term sealing performance and practical feasibility in routine endodontic practice.
